# Two complete mitochondrial genomes of *Paraleucogobio* fishes (Cypriniformes: Gobionidae)

**DOI:** 10.1080/23802359.2019.1623116

**Published:** 2019-07-10

**Authors:** Xia Zhang, Cuizhang Fu

**Affiliations:** Ministry of Education Key Laboratory for Biodiversity Science and Ecological Engineering, Coastal Ecosystems Research Station of the Yangtze River Estuary, Institute of Biodiversity Science and Institute of Eco-Chongming, School of Life Sciences, Fudan University, Shanghai, China

**Keywords:** Paraleucogobio, Gnathopogon, Gobionidae, phylogeny, *Paraleucogobio notacanthus*

## Abstract

Freshwater fishes in the genus *Paraleucogobio* include two species, *Paraleucogobio notacanthus* and *Paraleucogobio strigatus*. In this study, we determined complete mitochondrial genomes of *P. notacanthus* and *P. strigatus* to clarify their phylogenetic positions. The two mitochondrial genomes showed similar gene arrangements, codon use, gene overlaps or gene intervals with the length of 16,596 bp and 16,598 bp. Our phylogeny revealed that *P. notacanthus* and *P. strigatus* were nested within *Gnathopogon* fishes. The findings indicate that *Paraleucogobio* is a junior synonym of *Gnathopogon*.

Freshwater fishes in the genus *Paraleucogobio* include two species, *Paraleucogobio notacanthus* and *Paraleucogobio strigatus*, distributed in East Asia (Chen 1998). They are placed into the family Gobionidae according to an updated classification of the order Cypriniformes (Tan and Armbruster 2018). Previous molecular phylogeny of gobionid fishes showed that *P. strigatus* was a member of the genus *Gnathopogon* without examining the type species of *Paraleucogobio* (*P. notacanthus*) (Yang et al. 2006; Tang et al. 2011). In this study, we determined complete mitochondrial genomes of *P. notacanthus* and *P. strigatus* to clarify their phylogenetic positions.

Two sampled specimens were deposited in the Zoological Museum of Fudan University (FDZM), China, including *P. notacanthus* (voucher FDZM-PNQM20120327) from the Qimen, Anhui Province, China (29.93°N, 117.92°E) and *P. strigatus* (voucher FDZM-PSFC20170820) from the Fengcheng, Liaoning Province, China (40.46°N, 124.11°E). A high salt method was used to obtain total genomic DNA from muscle tissues (Miller et al. 1988). Complete mitochondrial genomes were obtained by Sanger sequencing, and assembled using the mitochondrial genome of *Gnathopogon imberbis* as reference (Gao et al. 2016).

The mitochondrial genomes of *P. notacanthus* and *P. strigatus* (GenBank accession numbers MK852686 and MK852687) were comprised of 13 protein-coding genes, two ribosomal RNA (rRNA) genes, 22 transfer RNA (tRNA) genes, and one control region with the lengths of 16596 bp and 16598 bp. The base compositions of two mitochondrial genomes had slightly A + T bias of 56.4% and 56.6%. The *ND6* and 8 tRNA genes were encoded on the light strand, and the remaining genes on the heavy strand. Among 13 protein-coding genes, ATG were used as start codon except for the COI gene using GTG, and there were four types of stop codons (TAA, TAG, TA–, and T–). Gene overlaps occurred among 6 pairs of adjacent genes with the overlapped size from 1 bp to 7 bp. Gene intervals were observed among 13 pairs of adjacent genes with the range from 1 bp to 32 bp. Similar patterns in gene arrangements, codon use, gene overlaps or gene intervals had also been reported in other mitochondrial genomes of gobionid fishes (Gao et al. 2016; Wu et al. 2018; Li et al. 2018).

Phylogenetic relationships between *Paraleucogobio* fishes and their relatives (Tang et al. 2011) were inferred under Mrbayes ver. 3.2.6 (Ronquist et al. 2012) using six partitions of 23 mitochondrial genomes, i.e. each codon of all protein-coding genes (excluding *ND6*), 12S rRNA gene, 16S rRNA gene and all tRNA genes (Figure 1). The results showed that *P. notacanthus* and *P. strigatus* were nested within *Gnathopogon* fishes. Our findings indicate that *Paraleucogobio* is a junior synonym of *Gnathopogon*, which would support earlier treatments of *P. notacanthus* and *P. strigatus* as members of *Gnathopogon* (Bănărescu and Nalbant 1967).

**Figure 1.**
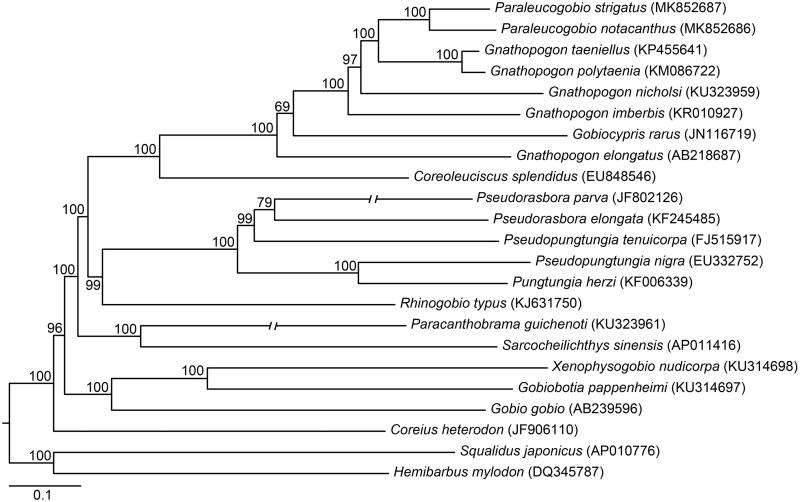
Topology of Bayesian tree for phylogenetic relationships between *Paraleucogobio* fishes and their relatives based on 23 mitochondrial genomes. Bayesian posterior probabilities are shown above or below branches for the Bayesian analyses. GenBank accession numbers are given in parentheses.
